# Characterization of New Recombinant Forms of HIV-1 From the Comunitat Valenciana (Spain) by Phylogenetic Incongruence

**DOI:** 10.3389/fmicb.2019.01006

**Published:** 2019-05-22

**Authors:** Beatriz Beamud, María Alma Bracho, Fernando González-Candelas

**Affiliations:** ^1^Instituto de Biología Integrativa de Sistemas, Consejo Superior de Investigaciones Científicas, Universitat de València, Valencia, Spain; ^2^Unidad Mixta de Investigación Infección y Salud Pública, Fundación para el Fomento de la Investigación Sanitaria y Biomédica de la Comunitat Valenciana, Universitat de València, Valencia, Spain; ^3^Área de Genómica y Salud, Fundación para el Fomento de la Investigación Sanitaria y Biomédica de la Comunitat Valenciana, Valencia, Spain; ^4^Centro de Investigación Biomédica en Red de Epidemiología y Salud Pública, Valencia, Spain

**Keywords:** HIV-1, nearly-full genome, recombination, phylogeny, CRFs, URFs

## Abstract

Recombination is one of the main processes shaping the evolution of HIV-1, with relevant consequences for its epidemiology. In fact, Circulating and Unique Recombinant Forms (CRFs and URFs) cause 23% of current infections. The routine analyses of antiretroviral resistance yield partial *pol* gene sequences that can be exploited for molecular epidemiology surveillance but also to study viral diversity and to detect potential recombinant samples. Among the *pol* sequences derived from a large sample dataset from the Comunitat Valenciana (Spain), we identified nine putative recombinant samples. We aimed at fully characterizing these samples and performing a detailed analysis of the corresponding recombination events. We obtained nearly full-genome sequences and used jpHMM and RDP4 to detect and characterize recombinant fragments. We assessed the confidence of these inferences by likelihood mapping and phylogenetic placement with topology congruence tests. Next, we performed a phylogenetic analysis of each putative recombinant fragment to determine its relationships to previously described recombinant forms. We found that two samples related to CRF44_BF whereas the rest corresponded to new URFs (two URF_AD, one URF_BG that can constitute a new CRF resulting from subtype B and CRF24_BG, and two URF_cpx composed of A, G, K, H, and J subtypes). These URFs have a complex recombination pattern that cannot be determined accurately. They seem to have arisen by successive recombination events among lineages, including other CRFs. Our results highlight the usefulness of routine surveillance analysis for the detection of new HIV-1 recombination forms and, at the same time, the need for full-genome sequencing and recombination detection guidelines to properly characterize this complex process.

## Introduction

The high genetic variability of HIV-1 is one of the main hurdles to control the current epidemic of this virus. This diversity is the result of high mutation (Abram et al., 2010), replication (Rodrigo et al., 1999), and recombination rates. The recombinogenic nature of HIV-1 is well-known, as this virus has one of the highest rates known (Rodrigo et al., 1999; Shriner et al., 2004). Recombination has important evolutionary consequences for viruses and has been associated with the expansion of viral host ranges, the emergence of new variants, increases in pathogenesis and virulence, the alteration of tropisms, the immune escape, and resistance to antivirals (Martin et al., 2011; Simon-Loriere and Holmes, 2011). In addition, recombination also plays an important role in HIV-1 epidemiology. Up to date, 91 circulating recombinant forms (CRFs) have been described (Los Alamos National Laboratory^1^). CRFs are recombinant HIV-1 genomes that share the same recombination breakpoints (BPs) between the same parentals and have been found in at least three non-epidemiologically related individuals. If a recombinant form does not fulfill the requirements to be considered a CRF, it is called a unique recombinant form (URF). RFs can also be classified according to their mosaic structure: they are denoted as complex (cpx) if they are composed by three or more subtypes or second-generation recombinants (SGRs) if they result from a recombination event in which pre-existing RFs are involved. RFs account for 23% of the current infections and are responsible for almost half of total infections in areas such as Central Africa (Hemelaar et al., 2019).

HIV-1 subtyping is still dominated by the partial sequencing of the *pol* gene used in routine analyses of antiretroviral resistance mutations. However, the development of whole genome sequencing (WGS) and its reducing costs have led to a substantial increase in the availability of complete HIV-1 genomes. This is partially responsible for the increased reporting of RFs world-wide. This increase also results from the mixing of newly formed HIV-1 variants, resulting in a complex and dynamic epidemiology (Hemelaar et al., 2019). Spain is one of the western European countries with the highest prevalence of HIV-1^2^. Subtype B is responsible for 82–88% of the infections in this country followed by RFs, which cause about 9–10% of cases (Yebra et al., 2012). In fact, the three CRFs described from Spanish samples so far, CRF14_BG, CRF47_BF, and CRF73_BG (Delgado et al., 2002; Fernández-García et al., 2010, 2016), involve the B subtype. Apart from these, only a few studies with complete HIV-1 genome sequences have been done in this country using non-B subtypes (Casado et al., 2003; Sierra et al., 2005; Holguin et al., 2008; Cuevas et al., 2010). Hence, the prevalence of recombinant forms in Spain, as in many other countries, might be underestimated (Yebra et al., 2012).

The *pol* sequences obtained in the analyses of resistance mutations can be used to gain insight into the population dynamics and evolution of the corresponding lineages. These analyses can also identify sequences that are not neatly included in any of the major subtypes or sub-subtypes of HIV-1. They may represent new variants or RFs, relevant for the epidemiological characterization of the viral population. There is no standard procedure to detect recombination in HIV-1 and different methods have been used so far (a comprehensive list of recombination analysis software^3^). Due to this lack of standardization and the intrinsic difficulties in identifying recombinant events, some authors have postulated that several methods should be used to obtain a precise picture of recombination (Posada, 2002). The hallmark of recombination is the presence of fragments with different ancestries in the same genome. Therefore, the ultimate test for recombination should be based on revealing a statistically significant lack of phylogenetic congruence among portions of the recombinant genomes (Pérez-Losada et al., 2015). In order to test the statistical significance of alternate topologies, the corresponding multiple alignments must incorporate adequate levels of genetic variation. Consequently, analyses of the phylogenetic signal should also be included in this procedure. This is most relevant for small recombinant regions in which an insufficient phylogenetic signal may lead to wrong inferences of phylogenies (Jia et al., 2014).

During routine analyses of resistance mutations in HIV-1 samples from the Comunitat Valenciana (Spain) (Patiño-Galindo et al., 2017), we detected several samples whose partial *pol* sequences did not cluster with high support in any sub-subtype. Our aim here is to describe their full genomic characterization and to perform an in-depth analysis of recombination using different methods and confirm them by means of phylogenetic congruence tests.

## Materials and Methods

### Samples

From 2010 to 2013, about 1,800 serum samples of individuals infected with HIV-1 were screened for antiretroviral resistance mutations in our laboratory (Patiño-Galindo et al., 2017). For this purpose, a partial region (1,302 nt) of the *pol* gene was sequenced. The phylogenetic analysis of this region revealed nine samples with a suspected recombinant nature because they did not cluster with high enough bootstrap support with reference sequences of pure subtypes and representative CRFs (data not shown). These nine samples, all from Spanish patients that attended hospitals of the Comunitat Valenciana (Spain), were subjected to nearly-WGS.

### Extraction, Amplification, and Sequencing

RNA was extracted from sera using the NucliSENS^®^ EasyMag^®^ automated platform (bioMérieux, Marcy, L’Étoile, France). Reverse transcription (RT) of RNA was performed in two adjacent genome regions to improve yield. The first hemigenome was obtained by priming with IN-T-A2 (Thomson et al., 2002) in the *vif* gene (nts: 5192–5218, HXB2, GenBank Accession No. K03455). The second hemigenome was obtained with UNINEF7′ (Nadai et al., 2008), close to the 3′ end of the viral RNA (nts: 9605–9632, HXB2). The conditions for RT and PCR were slightly modified from Nadai et al. (2008). RNA (4 μL) was reverse transcribed in a total volume of 20 μL with 500 uM dNTP, 2.5 uM primer, 1x RT buffer, 40 U RNasin^®^ (Promega, Madison, WI, United States), 10 mM DTT and 200 U of SuperScript^TM^ III reverse transcriptase (Invitrogen^TM^, Carlsbad, CA, United States). Reagents and RNA were incubated for cDNA synthesis at 50°C for 2 h, followed by 85°C for 5 min.

**FIGURE 1:**
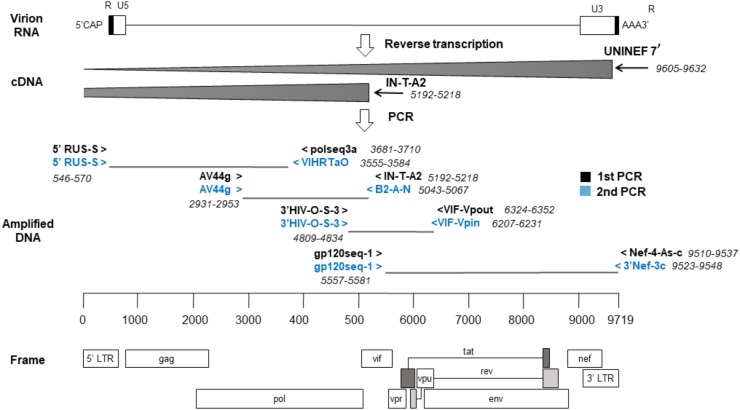
Sequencing strategy by the nearly full-length RT-PCR method. The locations of the primers used are relative to the reference genome, HXB2. cDNA synthesis was done in two reactions and a strategy of four overlapping amplicons was followed by PCR.

Next, four overlapping regions were amplified using first-round PCRs and, if needed, nested-PCRs (Figure 1). The first amplicon consisted of about 3 kb from 5′ LTR U5 to the middle of *pol* (nts: 571–3554, HXB2). The second amplicon, of 2.1 kb, covered from the second half of *pol* to the start of *vif* (nts: 2930–5042, HXB2). The third amplicon, of approximately 1.4 kb, covered until *env* gp160. Lastly, the fourth amplicon consisted of 3.9 kb from *env* gp160 to the end of *nef* (nts: 5582–9511, HXB2). The reaction volume was 25 μL, containing 1x PCR buffer, 350 uM dNTP mixture, 0.4 uM of each primer and 2.5 U TaKaRa Ex Taq^®^ (Takara Bio, Shiga, Japan). Cycling conditions were 94°C for 2 min and then 10 cycles of (94°C 10 s, 60°C 30 s, 68°C 3 min), and 20 cycles of (94°C 10 s, 55°C 30 s, 68°C 3 min) followed by 68°C for 10 min. Nested-PCRs were done with the same conditions as above but adding 1.5 μL of the previous PCR product with appropriate primers.

Positive PCR products were purified with a High Pure PCR Product Purification Kit (Roche Applied Science, Indianapolis, IN, United States) and sequenced using a BigDye Terminator v3.1 Cycle Sequencing Kit (Applied Biosystems, Foster City, CA, United States) in an ABI 3730xl DNA sequencer (Roche, Nutley, NJ, United States). The set of primers used in this study is available upon request. We obtained the consensus nearly full-length genome (8.9 kb) by assembling the sequence readings that passed the default quality filter of Pregap4 (Bonfield et al., 1995; Nadai et al., 2008) along with visual inspection of polymorphic regions. For genomes with more than one contig, scaffolding was done after comparison with the closest reference genome available at the Los Alamos National Laboratory (LANL) database. The final sequences have been deposited in the GenBank with Accession Nos. MK095228–MK095236.

### Recombination Analysis

The mosaic recombinant structure of the samples was screened using two different strategies. Firstly, breakpoints (BPs) were determined with the default jpHMM options (Schultz et al., 2010). Secondly, we obtained recombination events and BPs that were supported by at least three of the seven selected tests implemented in RDP4: RDP (Martin and Rybicki, 2000), GENECONV (Padidam et al., 1999), BootScan (Martin et al., 2005), MaxChi (Smith, 1992), Chimaera (Posada and Crandall, 2001), SiScan (Gibbs et al., 2000) and 3Seq (Boni et al., 2007). Default parameter values were used except for window size that was increased to 60 nt in RDP, to 120 in MaxChi and Chimaera, and to 500 in BootScan and SiScan, as suggested (Lemey et al., 2009).

For jpHMM we used the nine sequences obtained above. To avoid confounding effects between recombinant samples, we used a separate alignment for each sample in RDP4. These alignments consisted of one sequence of each ‘pure’ subtype and sub-subtypes A1, A2, F1, and F2 (from RIP Alignment^4^) and each putative recombinant sample using the option -add of MAFFT v.7 (Katoh and Standley, 2013).

After detecting recombination, we wanted to further contrast the confidence of each recombination event independently. For this, we used a slightly modified phylogenetic congruence testing pipeline. The corresponding BPs of each detection strategy delimited sequence regions that were extracted and independently aligned using MAFFT v.7 (Katoh and Standley, 2013) *(option –add*) with the RIP reference alignment detailed above. This partial alignment was used to reconstruct a reference maximum likelihood (ML) tree using IQ-TREE v.1.6.5 (Nguyen et al., 2015) with the best evolutionary model (GTR+F+I+G4) determined by the same software (Kalyaanamoorthy et al., 2017). To test the phylogenetic congruence of the recombinant fragments in each genome, we constructed all possible alternative trees by clustering each sample with all the (sub-)subtypes in the reference alignment. For this, we used the R packages ape (Paradis and Schliep, 2018) and phytools (Revell, 2011). The 11 alternative trees for each sample were used for Expected Likelihood Weights (ELWs) (Strimmer and Rambaut, 2002) topological congruence test with the partial alignment corresponding to each fragment. This test assigns relative support values to the alternative topologies based on the likelihood values of the corresponding alignments. These tests were performed with IQ-TREE v.1.6.5 using 10,000 RELL replicates. Additionally, the level of phylogenetic signal of each partial alignment was also assessed by means of likelihood mapping with the evaluation of 1,000 random quartets using IQ-TREE v.1.6.5. A proportion higher than 0.25 of unresolved quartets was considered to represent a lack of phylogenetic signal (Strimmer and Rambaut, 2002; Lemey et al., 2009).

Next, each fragment of the recombinant samples was further characterized by phylogenetic analysis with representative sequences of HIV-1, including RFs, as above. The alignment was obtained from LANL database (RIP v.2017, 145 sequences) but adding, with MAFFT v.7, all the URFs identified as such (resulting in a total of 226 sequences). Then, new ML trees were built with 1,000 bootstrap replicates. The closest relatives (CRs) of each fragment were determined considering as CRs the minimum set of sequences that grouped with the fragment of interest with a bootstrap support value higher than 70%.

Finally, the inferred mosaic structure from the above analyses was drawn using the ggplot2 library in R (Wickham, 2016). These analyses, except the execution of RDP4, have been integrated into a publicly available pipeline accessible at https://github.com/BBeamud/PhyloRecomb/.

## Results

We obtained nearly complete genome sequences of seven samples, but our sequencing strategy failed to yield enough information in two cases. Between 86 and 92% of the reference genome (HXB2) length was covered in our samples. The per base coverage ranged from 2.44 to 4.53. In four of these genomes, it was not possible to assemble all the sequencing reads into a single contig due to hypervariability in the *env* gene. This is reflected in the proportion of undetermined bases of each sequence that were used for scaffolding (Table 1). For the incomplete genomes, we obtained 41% (sample 678) and 49% (sample 3164) of the reference genome length with coverage depths of 1.64 and 1.66, respectively. Additional sequencing of these two samples was not possible due to lack of biological material.

**Table 1:** Summary statistics of the near complete genomes obtained for the 9 HIV-1 samples studied.

Sample	Total reads	Mean length of reads (nt)	Contigs	Ambiguities (N’s)	Assembly length (nt)	HXB2 coverage	Depth of coverage
79	55	740.49	1	25	8987	0.92	4.53
93	60	540.03	2	354	8922	0.87	3.66
678	10	665.30	1	4	4016	0.41	1.66
703	47	586.17	1	0	8994	0.91	3.06
724	53	558.04	1	84	9012	0.90	3.29
2011	54	613.93	2	396	8852	0.86	3.92
2104	35	599.49	2	352	8934	0.88	2.44
3011	33	694.88	1	0	8961	0.91	2.56
3164	9	883.00	2	2538	7379	0.49	1.64

Recombinant mosaic structures were obtained by two strategies. Firstly, we considered the recombination events and their defining BPs supported by at least three of the methods tested in RDP4. Additionally, we considered the BPs reported by jpHMM. These two programs assign a subtype for each fragment. All the strategies revealed putative recombination events in all the samples, but the total number of such events differed among them. To facilitate the comparison between detection strategies, we retained as major parental (MP) the subtype with most genomic nucleotides assigned. In addition, we considered the same recombination event if the minor assigned subtype and the inferred BPs were similar ( ± 200 nt) in both sides for both strategies. A total of 42 recombination events were detected but only eight were shared by the two strategies. Of the 34 remaining events, 21 were detected exclusively by jpHMM and 13 by RDP4. There were substantial differences in the performance of the methods implemented in RDP4, with a minimum of 3 (GENECONV) and a maximum of 19 (MaxChi and 3Seq) detected events (Table 2). Shared events had an average length of 1454.9 nt with a difference in the start and end positions of 14.9 and 59.0 nt, respectively. The shared events detected by RDP4 were on average 11.9 nt shorter than those detected by jpHMM. On the contrary, unique events by jpHMM and RDP4 had lengths of 513.6 and 1399.4 nt. Surprisingly, some large events detected by RDP4 were not shared with jpHMM. This was due to RDP4 failing to detect several events of the minor subtypes and reporting them as unique, larger recombination events (Table 3, samples 2011, 2104, 3011, and 3164).

**Table 2:** Support of each putative recombination event detected by the different methods implemented in RDP4.

Sample (MP)^a^	Event	Subtype	Start	End	RDP	GENECONV	Bootscan	Maxchi	Chimera	SiSscan	3Seq
79 (F1)	1^∗∗^	B	2470	3707	2.23E-09	NS	3.21E-11	8.77E-07	3.87E-07	NS	4.88E-14
93 (F1)	1^∗∗^	B	2471	3708	5.38E-09	NS	1.92E-11	3.26E-07	7.95E-08	NS	4.88E-14
678 (G)	1^∗∗^	B	2592	4802	3.60E-16	NS	1.31E-16	6.21E-19	5.28E-22	2.98E-12	5.36E-39
703 (G)	1^∗∗^	B	2558	4803	1.13E-38	8.23E-04	8.96E-31	1.92E-17	7.25E-21	1.34E-15	4.88E-14
	2R	U(D)	8697	8860	NS	NS	NS	9.82E-06	2.91E-03	NS	2.07E-02
724 (G)	1^∗∗^	B	2559	4804	1.29E-39	1.11E-03	1.54E-40	1.29E-18	6.97E-22	3.22E-17	4.88E-14
	2R	H	7337	7612	1.75E-03	1.77E-03	NS	3.70E-03	8.03E-04	NS	NS
2011(A1)	1^∗∗^	D	2161	3069	6.96E-08	NS	9.57E-04	2.12E-04	1.78E-07	NS	6.31E-10
	2R	U(F)	3070	5923	4.30E-02	NS	NS	6.10E-04	9.32E-03	5.03E-10	2.44E-14
	3R^∗∗^	D	6077	9384	6.13E-06	NS	2.42E-08	1.05E-05	7.31E-10	7.91E-09	8.30E-13
2104 (A1)	1^∗∗^	D	2135	3252	7.84E-09	NS	3.86E-03	3.16E-08	5.42E-09	NS	7.25E-12
	2R	U(F2)	3253	5924	NS	NS	NS	1.36E-05	2.66E-02	1.41E-11	2.44E-14
	3R^∗∗^	D	6078	9384	5.51E-04	NS	1.25E-05	1.02E-12	5.99E-12	9.09E-08	2.44E-14
3011 (A1)	1R	C	1526	1989	1.47E-02	NS	1.58E-02	6.05E-03	NS	NS	1.43E-02
	2R	U(F1)	2169	3238	NS	NS	NS	1.34E-02	4.64E-02	NS	1.49E-05
	3R	F2	3449	5662	NS	NS	NS	8.89E-03	7.98E-03	NS	5.90E-05
	7^∗∗^	K	6109	6486	1.19E-05	NS	1.96E-05	1.72E-03	4.07E-04	NS	4.56E-02
	4R	U(C))	6608	6847	2.39E-02	NS	NS	NS	NS	4.11E-04	7.59E-03
	5R	U(F2)	8335	9385	NS	NS	NS	1.03E-04	1.22E-05	4.29E-09	NS
3164 (A1)	1R^∗^	K	2727	3031	7.20E-04	NS	1.51E-03	NS	NS	NS	2.70E-03
	2R	F2	6079	6345	1.74E-07	NS	2.16E-05	1.32E-04	1.92E-05	NS	3.69E-05

*Asterisks indicate events confirmed by ELW (best topology) and phylogenetic signal (unresolved quartets, UQ < 0.25). ^∗^Supported by ELW; ^∗∗^Supported by ELW + Phylogenetic signal. ^a^Major parental.*

**Table 3:** Recombination events detected by jpHMM and RPD4 and phylogenetic analysis of the studied samples.

	jpHMM						
Sample (MP)^a^	Event	Subtype	Start	End	ELW	UQ	# CRs (taxa)
79 (F1)	1^∗∗^	B	2466	3700	B	0.067	1 (44_BF)
93(F1)	1^∗∗^	B	2467	3701	B	0.068	1 (44_BF)
678 (G)	1^∗∗^	B	2576	4830	B	0.037	1 (B)
703(G)	1^∗∗^	B	2575	4829	B	0.032	1(B)
724(G)	1^∗∗^	B	2576	4830	B	0.027	1 (B)
2011 (A1)	1^∗∗^	D	2135	3254	D|b	0.115	5 (D)
	2J^∗∗^	D	6077	7317	D	0.044	4 (D)
	3J^∗∗^	D	7602	8821	D	0.064	20 (D)
	4J	B	8822	9199	B|D|H|A2	0.109	19 (D)
	5J^∗∗^	D	9200	9411	D|b	0.072	12 (D)
2104 (A1)	1^∗∗^	D	2137	3255	D|b	0.123	1 (19_cpx)
	2J^∗∗^	D	6079	8818	D	0.018	4 (D)
	3J	B	8820	9200	B|H|D|A2	0.117	1 (03_AB)
	4J^∗∗^	D	9202	9412	D|b	0.078	14 (D)
3011 (A1)	1J^∗∗^	G	1628	2287	G	0.139	1 (13_cpx)
	2J	J	2691	3116	J|K	0.252	2 (27|13_cpx)
	3J^∗^	H	3118	3224	H|a1|a2	0.553	4 (H)
	4J	B	3483	4147	K|F2|F1|G	0.270	1 (04_cpx)
	5J^∗^	G	4149	4607	G	0.337	1 (27_cpx)
	6J	B	5085	5706	K|J	0.104	3 (URF_U, 09_cpx, URF_0209)
	7^∗∗^	K	6076	6296	K	0.149	1 (K)
	8J^∗^	H	8335	8558	H	0.301	1 (04_cpx)
	9J^∗^	J	8560	8670	J|c|k	0.431	1 (URF_A1D)
	10J^∗^	H	8867	9018	H	0.269	H
3164 (A1)	1J^∗∗^	K	6079	6298	K	0.156	1 (K)
	2J^∗^	H	8336	8559	H	0.284	1 (04_cpx)
	3J^∗^	J	8561	8663	J|c	0.456	2 (45_cpx, URF_U)
	4J^∗^	H	8870	8998	H	0.376	4 (H)
	5J	B	9000	9299	H|B|F1|F2|J|K|G|D	0.149	222 (HIV-1)

**RDP4**							
**Sample (MP)^a^**	**Event**	**Subtype**	**Start**	**End**	**ELW**	**UQ**	**# CRs (taxa)**

79 (F1)	1^∗∗^	B	2470	3707	B	0.057	1 (44_BF)
93 (F1)	1^∗∗^	B	2471	3708	B	0.067	1 (44_BF)
678 (G)	1^∗∗^	B	2592	4802	B	0.026	1 (51_01B)
703 (G)	1^∗∗^	B	2558	4803	B	0.028	1 (B)
	2R	U(D)	8697	8860	J|K|B|D|H|C	0.388	3 (24|23|20_BG)
724 (G)	1^∗∗^	B	2559	4804	B	0.034	222 (HIV-1)
	2R	H	7337	7612	H|G|J	0.281	3 (24|23|20_BG)
2011(A1)	1^∗∗^	D	2161	3069	D|b	0.147	5 (D)
	2R	U(F)	3070	5923	A1	0.013	1 (A3)
	3R^∗∗^	D	6077	9384	D	0	11 (D)
2104 (A1)	1^∗∗^	D	2135	3252	D|b	0.154	5 (D)
	2R	U(F2)	3253	5924	A1	0.02	1 (A3)
	3R^∗∗^	D	6078	9384	D	0	17 (D)
3011 (A1)	1R	C	1526	1989	G|C	0.195	2 (32_06A1, 06_cpx)
	2R	U(F1)	2169	3238	H|K|J	0.137	1 (27_cpx)
	3R	F2	3449	5662	K|C|J	0.034	1 (27_cpx)
	7^∗∗^	K	6109	6486	K	0.125	1 (39_BF)
	4R	U(C)	6608	6847	A2|A1|H|F2	0.396	1 (URF_U)
	5R	U(F2)	8335	9385	H	0.026	5 (H)
3164 (A1)	1R^∗^	K	2727	3031	K	0.307	1 (49_cpx)
	2R	F2	6079	6345	K	0.172	2 (K)

*Events detected by jpHMM and RDP4 are named with one single number and those detected by only one method are named with a number and a letter (J = jpHMM, R = RDP4). Asterisks indicate events confirmed by ELW (best topology) and phylogenetic signal (unresolved quartets, UQ < 0.25). Alternative topologies found by ELW (less than 0.5 difference with the best topology) are shown in lowercase. The number of closest relatives (CRs) and corresponding pure subtype or RF are shown in the last column. ^a^Major parental; ^∗^Supported by ELW; ^∗∗^Supported by ELW + Phylogenetic signal.*

**FIGURE 2:**
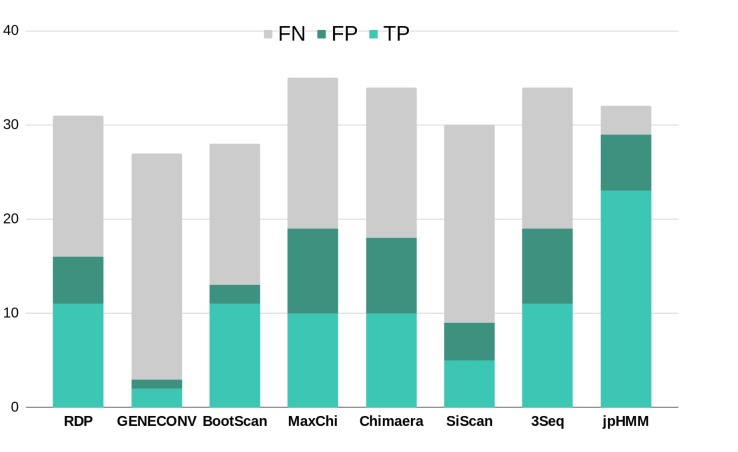
Summary of true positive (TP), false positive (FP) and false negative (FN) recombination events detected by the different methods used in this study according to the results of ELW tests. RDP, GENECONV, BootScan, Maxchi, Chimaera, SiScan, and 3Seq were analyzed with RDP4.

To further corroborate these events, we performed ELW congruence tests and likelihood mapping analyses for each fragment. A recombination event was considered verified only when the tree with the sample fragment grouped with the minor subtype identified had a relative support value, as determined by ELW, larger than 0.5 than that of the second-best tree. Additionally, we considered fragments with less than 0.25 unresolved quartets reliable for phylogenetic inferences. All the events jointly detected by jpHMM and RPD4 were verified by ELW tests and phylogenetic signal. However, 15 out of the 21 events detected only by jpHMM were corroborated, regardless of their phylogenetic signal, whereas only 3 of the 13 events detected only by RDP4 were verified. For the latter, the main discrepancies were observed in the assigned subtype whereas for jpHMM a high proportion of cases with low phylogenetic signal was observed (Table 3). A summary of true positive, false positive and false negative recombination events, detected by the different methods implemented in RDP4 and jpHMM, is shown in Figure 2. The overall best performance corresponds to jpHMM, with 23 corroborated events of the 29 detected with this method. The worst performing method was GENECONV with only two corroborated events of the three detected.

Different mosaic structures were observed in the samples considering verified events. In particular, we observed two BF recombinants, two AD recombinants, three BG recombinants, and two complex recombinant forms. These mosaic structures were refined with the reconstruction of ML trees for each fragment based on the analysis of closest relatives (CRs) with 226 representative sequences of HIV-1. We performed these analyses for all the major and minor parental fragments although the results of the former analyses are not shown for simplification (Table 3).

**FIGURE 3:**
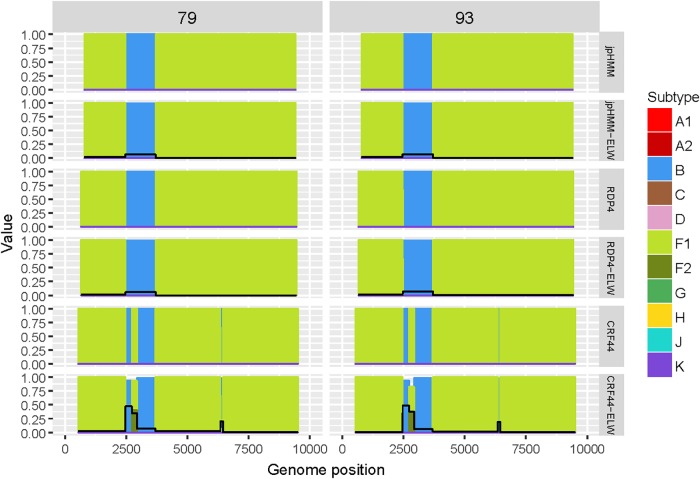
Recombination analyses of samples 79 and 93 with recombination BPs determined by different sources (jpHMM, RDP4 and CRF44_BF reference BPs) and phylogenetic congruence (ELW) tests. The black line represents the proportion of unresolved quartets, which correspond to the inverse of the phylogenetic signal.

The two BF recombinants corresponded to samples 79 and 93. They showed the same pattern and similar BPs by both jpHMM and RDP4. A single recombination event of subtype B was detected and verified in the region located between nucleotides 2,466 and 3,708 (all the positions reported are referred to HXB2). This structure resembles that of CRF38_BF (Ruchansky et al., 2009) although the CRs analyses of the recombinant fragments of samples 79 and 93 showed that they are related to CRF44_BF (Delgado et al., 2010) (Table 3). The latter has three recombination events (all of subtype B) that were not detected by jpHMM nor RDP4. We re-analyzed samples 79 and 93 by performing congruence analyses with the reference breakpoints of CRF44_BF^5^ to confirm it relatedness. The results of the ELW tests showed a lack of congruence for the three recombination events with their grouping with subtype B, thus corroborating them (Figure 3). Hence, the CRs analyses allowed us to classify samples 79 and 93 as CRF44_BF, despite their initial identification as CRF38_BF.

**FIGURE 4:**
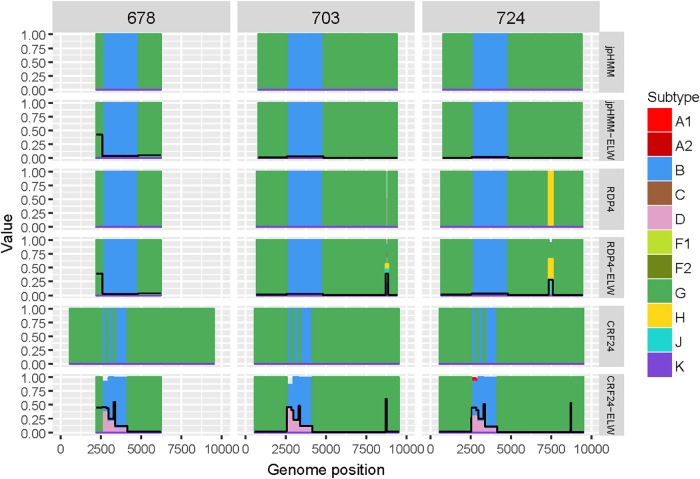
Recombination analyses of samples 678, 703, and 724 with recombination BPs determined by different sources (jpHMM, RDP4, and CRF24_BG reference BPs) and phylogenetic congruence (ELW) tests. The black line represents the proportion of unresolved quartets, which correspond to the inverse of the phylogenetic signal.

Samples 678, 703, and 724 are BG recombinants, with G being the major parental with a single B recombination event. A similar situation to that in BF recombinants was found in this group, except for two recombination events detected only by RDP4, 703_2R (U|D) and 724_2R(H), which were not verified by ELW and phylogenetic signal tests. CRs analyses showed that the G fragment was related to CRF24_BG whereas the B fragment grouped consistently with pure B sequences or CRF51_01B (Table 3). To confirm that samples 678, 703, and 724 were indeed CRF24_BG we proceeded as above, with the BPs defined at the LANL database for this CRF. In this case, the results of the ELW tests did not reveal four different B recombination events, as in CRF24_BG, but recognized only the single B event detected jointly by RDP4 and jpHMM (Figure 4). It is important to note that in the case of sequence 678, the comparisons were limited because only a partial genome was obtained.

**FIGURE 5:**
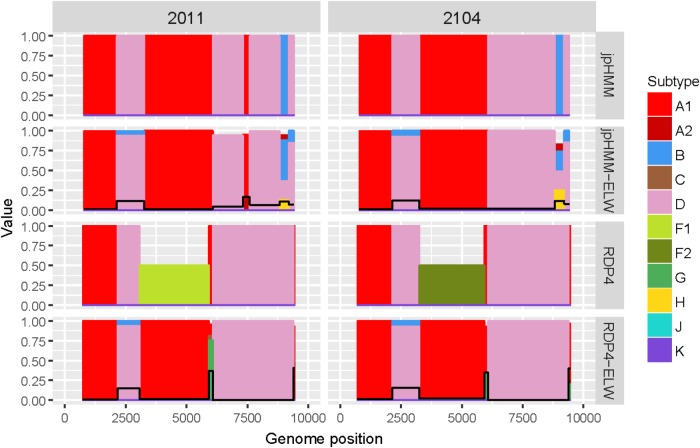
Recombination analyses of samples 2011 and 2104 with recombination BPs determined by different sources (jpHMM and RDP4) and phylogenetic congruence (ELW) tests. Values for inconclusive results by RDP4 are arbitrarily represented as 0.5. The black line represents the proportion of unresolved quartets, which correspond to the inverse of the phylogenetic signal.

The two AD recombinants corresponded to samples 2011 and 2014 with A subtype being the major parental and with four and three fragments of subtype D, respectively. Initially, more recombination events of other subtypes were identified by jpHMM and RDP4. However, they were discarded based on the results of the ELW congruence tests. RDP4 failed to detect three events that were detected by jpHMM and further verified by tree-topology and phylogenetic signal (Figure 5). CRs analyses showed that the A portion corresponded to sub-subtype A3 whereas the D fragments clustered with D ‘pure’ or CRF19_cpx sequences (Table 3). Despite small discrepancies, 2011 and 2014 present a similar recombination pattern but, to our knowledge, no similar pattern has been reported to the databases yet.

**FIGURE 6:**
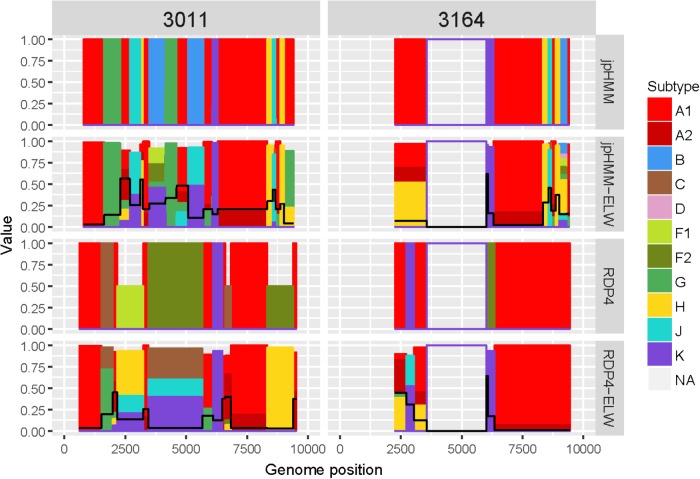
Recombination analyses of samples 3011 and 3164 with recombination BPs determined by different sources (jpHMM and RDP4) and phylogenetic congruence (ELW) tests. Values for inconclusive results by RDP4 are arbitrarily represented as 0.5. The black line represents the proportion of unresolved quartets, which correspond to the inverse of the phylogenetic signal.

The genome sequences derived from samples 3011 and 3164 were identified as complex forms, composed by more than two ‘pure’ subtypes: A, G, H, J, and K for 3011 and A, H, J, and K for 3164. For both samples, the A subtype was considered as the major parental although, in this case, there is little difference between the portion of the genome covered by the different subtypes. As before, the description of 3164 is limited because about only half of its genome sequence was obtained. These recombinant forms were very complex both in the detection and verification results (Figure 6). A total of 16 and seven different recombination events were detected for samples 3011 and 3164, respectively. JpHMM detected 10 events in sample 3011, which were different from the five events detected by RDP4. Only one event, 3011_7(K), was detected simultaneously by both methods (Table 3). None of the seven events detected in sample 3164, five by jpHMM and two by RDP4, was shared by the two methods.

Most of the events detected by jpHMM in sample 3011 (7/11) showed a low phylogenetic signal, with more than 25% of unresolved quartets (Table 3) whereas only one event detected by RDP4 in this sample failed this test. A similar result was observed for sample 3164, with three of the five events detected by jpHMM presenting low phylogenetic signal and with one of the two events detected by RDP4 also failing this test. This low signal might also affect subtyping inferences, with several discrepancies between the subtypes identified by jpHMM or RDP4 and those inferred as most likely by the phylogenetic congruence analyses (Table 3). Finally, the results of CRs analyses presented several cases of unclear relatedness. For instance, fragments of the major subtype of these two samples grouped within clade A but also with distinct URFs and complex forms, such as CRF45_cpx. The G and J fragments grouped with other complex forms, such as CRF13_cpx and CRF27_cpx. Finally, recombinant fragments of subtype H clustered with ‘pure’ H sequences or CRF04_cpx and those of subtype K were related to K ‘pure’ sequences (Table 3). Altogether, the mosaic structure of these genomes could not be determined unambiguously. Samples 3011 and 3164 showed important discrepancies between them and at present, no CRFs_cpx with these patterns has been reported to the databases.

## Discussion

In this study, we have described seven nearly-complete and two partial genomes of different HIV-1 recombinant forms, detected in infected individuals from Comunitat Valenciana (Spain). We have performed detailed analyses to characterize their recombination events. Firstly, we identified putative recombinant fragments using different methods, jpHMM and seven of those included in RDP4. In a second phase, we corroborated the recombinant fragments by evaluating their phylogenetic signal, testing their topological congruence and analyzing their closest relatives using a comprehensive dataset of HIV-1 sequences including representatives of all the subtypes, CRF and URFs.

Two samples, 79 and 93, were initially identified as CRF38_BF based on the results of jpHMM and RDP4. However, our additional analyses of closest relatives and ELW congruence test revealed that both were related to CRF44_BF, a recombinant form first described in Chile in 2010 (Delgado et al., 2010), but already circulating in Madrid (Spain) in 2005 to 2007 (Gonzalez-Alba et al., 2011). These samples were obtained in 2012 and 2013, thus indicating that this recombinant form was actively circulating in Spain a few years later. BF recombinants might be a larger family than previously thought with differences in the location of breakpoints or length of the inserted fragments (Cevallos et al., 2017). This hypothesis is confirmed in this study, providing two additional BF genomes that were not properly characterized based on the breakpoints reported by two detection methodologies but identified with additional analyses. Subsequent mutations or differences in methodology might prevent the detection of the actual mosaic structure of additional recombinant forms in this family. Thorough phylogenetic analyses, including congruence tests, should always be performed to reliably infer the recombination structure of HIV-1 samples.

The three BG recombinants shared a similar mosaic structure, which was further verified in our subsequent analyses. They might constitute a SGR resulting from a new recombination event between CRF24_BG and subtype B. CRF24_BG was firstly identified in Cuba in 2007 (Sierra et al., 2007; Cevallos et al., 2017). However, it was found in several Spanish individuals in Madrid a few years ago (Gonzalez-Alba et al., 2011). This, along with the high prevalence of subtype B and the origin of two other BG recombinant forms, CRF14_BG and CRF73_BG (Delgado et al., 2002; Fernández-García et al., 2016), in the Iberian Peninsula, suggests a scenario that fits well with the previous hypothesis as well as with the subsequent formation of new RFs with these subtypes.

Samples 2011 and 2014 might represent a new URF_AD, originated from several recombination events of ‘pure’ A3 and D sequences. In Spain, A and D subtypes are not frequent as they represent less than 1% of total infections (García et al., 2011; Gonzalez-Alba et al., 2011; Yebra et al., 2012; Patiño-Galindo et al., 2017) and no CRF_AD has been reported in this country so far. Hence, it is likely that this URF_AD has emerged in an endemic area for these subtypes and arrived in Spain by immigration.

Lastly, samples 3011 and 3164 represent complex forms composed of A, G, K, H and J subtypes that could not be identified accurately. Most of these subtypes, except G and A, are extremely rare in Spain (García et al., 2011; Gonzalez-Alba et al., 2011; Yebra et al., 2012; Patiño-Galindo et al., 2017). More specifically, in the Comunitat Valenciana, a previous analysis of the partial *pol* sequences of the 1,800 samples at the origin of this study revealed an important number of non-B infections (16.2%) but they did not include subtypes K, H, or J (Patiño-Galindo et al., 2017). These new URFs are an example of the enormous complexity of the recombination process in HIV-1 as several CRF_cpx are involved. The presence of CRFs from West-Central Africa, such as CRF9_cpx, CRF13_cpx, CRF27_cpx or CRF45_cpx, previously reported in Spain (Niama et al., 2009), might reflect an increased prevalence and generation of subsequent unique mosaic viruses in diverse geographical areas.

There are substantial differences in the performance of the eight methods used to detect recombination (Figure 2). The combined use of jpHMM and some (in our case at least three) methods implemented in RDP4 improves the reliable detection of recombination events: all the common events were further corroborated by ELW and phylogenetic signal analyses. This result agrees with previous suggestions of using several, methodologically different approaches to detect recombination events in HIV-1 (Posada, 2002). For those cases in which an event is not detected by one methodology, but it is positive according to others, additional corroboration analyses should be performed. In this case, 18 events detected by only jpHMM or RDP4 were later corroborated by ELW and phylogenetic signal analyses.

In general, RDP4 and jpHMM performed well for samples with one single recombination event. However, more false positive and negative events as well as cases of subtype misidentification were found for both programs with increasing complexity of the recombinant structures. Both methods showed the largest number of discrepancies in the detection of complex forms. The difficulties for the precise identification of the origin of these fragments were also evidenced by unresolved results in the congruence tests. Because some of these fragments are very short, they may not have enough phylogenetic signal to differentiate between subtypes. This poses problems for the two methods used to detect them. In addition, the paucity of complete sequence information about ‘rare’ subtypes makes the detection and verification of recombinants, especially those involving complex forms, a difficult task.

## Conclusion

We have identified nine recombinant forms circulating in the Comunitat Valenciana (Spain). Two of them are related to CRF44_BF but the rest correspond to new URFs. Some samples of this study revealed a complex recombination nature and origin. This might suggest that, apart from the endemic areas that exhibit the highest levels of genetic diversity and therefore represent recombination hotspots, other regions could be sources of recombinant progeny due to the increasing global gene flow (Foster et al., 2014). To better understand the viral diversity and dynamics, as well as the real impact of recombination on them, more efforts should be devoted to obtaining complete genome sequences and to perform in-depth analyses of recombination.

The present study highlights the utility of surveillance analyses of HIV-1 for additional goals to identify resistance mutations, such as studying genetic diversity and characterizing new recombinant forms. A thorough analysis of partial genome sequences obtained in the surveillance of antiretroviral resistance mutations may provide the best starting point for the discovery and characterization of new recombinant forms in HIV-1.

## Ethics Statement

Approval of surveillance studies by an ethics committee is not required as per local legislation and host institution (FISABIO) guidelines.

## Author Contributions

The idea for this study was conceived by BB, MAB, and FG-C, who was also in charge of supervising the whole project. Data collection and reference searching were done by BB and MAB. The data analyses and drafting the manuscript were done by BB. The final manuscript was written, curated, and confirmed by BB, MAB and FG-C.

## Conflict of Interest Statement

The authors declare that the research was conducted in the absence of any commercial or financial relationships that could be construed as a potential conflict of interest.

## Footnotes

1 http://www.hiv.lanl.gov

2 http://apps.who.int/gho/data/node.main.620?lang=en

3 http://bioinf.man.ac.uk/robertson/recombination/programs.shtml

4 https://www.hiv.lanl.gov/content/sequence/NEWALIGN/align.html#RIP

5 https://www.hiv.lanl.gov/content/sequence/HIV/CRFs/breakpoints.html#CRF44
